# Relationship between the updated oxygen reserve index and arterial partial pressure of oxygen: a prospective observational study

**DOI:** 10.1186/s40981-025-00796-7

**Published:** 2025-06-16

**Authors:** Hidemi Ishido, Keisuke Yoshida, Tsuyoshi Isosu, Shinju Obara, Satoki Inoue

**Affiliations:** 1 Department of Anesthesiology, School of Medicine, Fukushima Medical University, 1 Hikariga-OkaFukushima Prefecture, Fukushima City, 960-1295 Japan; 2 Department of Anesthesiology, Minami Tohoku Fukushima Hospital, Fukushima, Japan

**Keywords:** Oxygen reserve index (ORi), Arterial partial pressure of oxygen (PaO_2_), Hyperoxia, Hyperoxemia, Revision M

## Abstract

**Introduction:**

The oxygen reserve index (ORi™), a non-invasive variable that continuously reflects oxygenation, was first reported in 2016. With the 2018 update of ORi, the scaling between 0.00 and 1.00 was modified. This article provides a follow-up report on the relationship between the updated ORi and arterial partial pressure of oxygen (PaO_2_), based on our previous study using the original version of ORi.

**Methods:**

The updated ORi version analyzed in the present study used a Revision M sensor. Twenty adult patients who were scheduled for surgery under general anesthesia with arterial catheterization were enrolled. After induction of general anesthesia, arterial blood gas analysis was performed with the fraction of inspiratory oxygen (FiO_2_) set at 0.33. The PaO_2_ and ORi at the time of blood collection were recorded. After that, FiO_2_ was changed to achieve an ORi of around 0.5, 0.2, or 0, followed by arterial blood gas analysis. The relationship between ORi and PaO_2_ was then investigated using the data obtained.

**Results:**

Seventy-six datasets from the 20 patients were analyzed. When PaO_2_ was < 240 mmHg (*n* = 73), linear regression analysis showed a relatively positive correlation (*r*^2^ = 0.4683). The cut-off ORi value obtained from the receiver operating characteristic curve to detect PaO_2_ ≥ 150 mmHg was 0.45 (sensitivity 0.833, specificity 0.810). Four-quadrant plot analysis demonstrated that ORi has good trending ability with respect to PaO_2_ (concordance rate was 100.0%).

**Conclusion:**

Although the original and updated versions of ORi demonstrate similar properties regarding their ability to track PaO_2_ changes, the updated version has a wider absolute value range. Therefore, caution is warranted when interpreting ORi values, as absolute values may vary significantly between versions, even at the same PaO_2_ level.

## Introduction

The oxygen reserve index (ORi™) (Masimo Corp., Irvine, CA, USA) is a variable that reflects real-time oxygenation reserve status in the mild hyperoxic range; specifically, an arterial partial pressure of oxygen (PaO_2_) of approximately 100–200 mmHg. ORi is measured noninvasively using a multi-wavelength pulse co-oximeter adhesive sensor. The original ORi was measured with Revision L sensor and expressed as a nondimensional index ranging from 0.00 (no reserve) to 1.00 (maximum reserve) reflecting the oxygenation reserve status [1, 2]. To date, several studies have investigated the clinical utility of ORi in perioperative settings. Currently, the usefulness of ORi in rapid sequence induction of general anesthesia [3], pediatric anesthesia [4], and one-lung ventilation [5, 6] has been demonstrated and applied clinically.

The original ORi measured with the Revision L sensor had a limitation: it exhibits a plateau around 0.5 irrespective of large differences in PaO_2_, which means it cannot reflect oxygenation reserve precisely, even under maximum oxygenation [3, 7]. To address this limitation and enable a more precise representation of hyperoxemia, ORi was updated by introducing the Revision M sensor in 2018 and the Revision O sensor in 2019, both enabling a more precise scaling from 0.00 to 1.00. According to the manufacture, while the Revision O sensor improves trend tracking of percutaneously measured hemoglobin concentration (SpHb) compared to the Revision M sensor, it does not alter ORi values.

However, the relationship between the updated ORi and PaO_2_ have not been reported in detail, highlighting the necessity of reexamining their correlation. If the characteristics of ORi differ depending on the version, its interpretation must also be reconsidered. The aim of the present study is to investigate the characteristics of the updated ORi measured using the Revision M sensor, focusing particularly on the relationship between ORi and PaO_2_.

## Materials and methods

### Study design

This prospective study was conducted as a clinical trial, employing the same design as our previous study, which investigated the relationship between ORi and PaO_2_ using the original version of ORi (rainbow® sensor, R2–25, Revision L, Masimo Corp.) [7]. This study was approved by the Research Ethics Committee of Fukushima Medical University (No.2022–127) and registered at the Japan Registry of Clinical Trials (jRCT1022220035) before the enrollment of the first patient. Written informed consent was obtained from all individual patients.

### Patients

Twenty patients who were scheduled for surgical procedures under general anesthesia between February 2023 and December 2024 were enrolled. The inclusion criteria were as follows: (1) age of ≥ 20 years; (2) an American Society of Anesthesiologists physical status classification of 1 or 2; and (3) requirement for arterial catheter insertion during surgery for continuous blood pressure monitoring and/or arterial blood gas analysis. The exclusion criteria were as follows: (1) inability to wear the sensor due to finger deformity; (2) inadequate signal due to hypoperfusion of the fingers; (3) presence of cardiac or pulmonary disease (e.g., chronic obstructive pulmonary disease, interstitial pneumonia); and/or (4) preoperative anemia due to hemoglobinopathies (e.g., sickle cell disease, thalassemia).

### Data collection

After the patient entered the operating room, an ORi sensor (RD rainbow Lite SET® Sensor, Revision M, Masimo Corp.) was attached to a finger or toe. The sensor was shielded from ambient light. SpO_2_ and ORi values were displayed on a Root® monitor (software: V2.1.4.6.i) with Radical-7® (software: v1.6.2.0.i, Board: MX-5 7e23, Masimo Corp.).

**Fig. 1:**
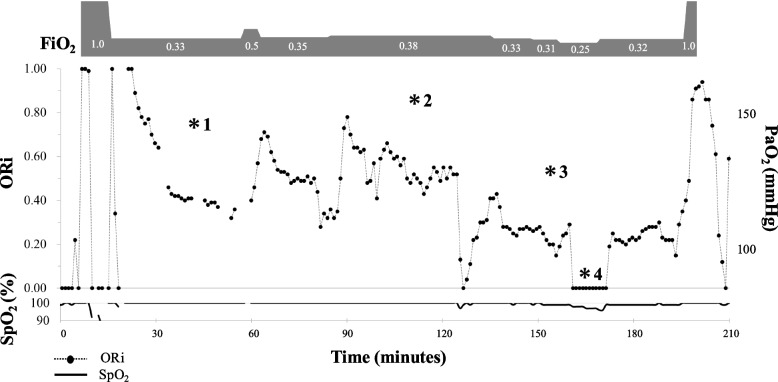
Trends of ORi during the experimental period in a 61-year-old female patient (height, 163 cm; weight, 73 kg) who underwent modified radical hysterectomy due to endometrial cancer. FiO_2_ (top) was set at 0.33 after induction of general anesthesia and PaO_2_ was measured approximately 10 min after stabilization of ORi (*1) (middle). Subsequently FiO_2_ was adjusted so as to achieve an ORi of 0.5, followed by another PaO_2_ measurement (*2). FiO_2_ was further decreased, and PaO_2_ was measured at an ORi of 0.2 (*3) and 0.0 (*4). SpO_2_ was maintained at ≥ 96% throughout the experimental period (bottom). Time 0 corresponds to the induction of general anesthesia. *ORi, oxygen reserve index; FiO*_*2*_*, fraction of inspiratory oxygen; PaO*_*2*_*, arterial partial pressure of oxygen; SpO*_*2*_*, peripheral oxygen saturation*

An arterial catheter was inserted into the radial artery after induction of general anesthesia. The anesthesia technique was selected according to the attending anesthesiologist’s discretion. General anesthesia was induced with propofol, remifentanil, fentanyl, and rocuronium, and maintained with either propofol, sevoflurane, or desflurane, along with remifentanil, fentanyl, and rocuronium. After tracheal intubation and the establishment of all necessary venous and arterial lines were completed, fraction of inspiratory oxygen (FiO_2_) was set at 0.33 with oxygen 0.5 L/min and air 2.5 L/min. Arterial blood gas analysis was performed approximately 10 min after ORi stabilization (Fig. 1). PaO_2_ was obtained by co-oximetry (SIMENS RAPIDLAB®1265, Siemens Healthcare Diagnostics Inc., Deerfield, IL, USA), and the values along with the corresponding ORi at the time of blood collection were recorded. Subsequently, FiO_2_ was adjusted to achieve an ORi of approximately 0.5, followed by arterial blood gas analysis approximately 10 min after stabilization of ORi. This procedure was repeated with an ORi target of 0.2. If the PaO_2_ at this stage was < 100 mmHg, the measurement for the current study was terminated and the FiO_2_ was set by the attending anesthesiologist based on peripheral oxygen saturation (SpO_2_) to avoid hypoxemia. Otherwise, FiO_2_ was adjusted to achieve an ORi of approximately 0.0, followed by arterial blood gas analysis and recording of ORi. The measurement was then terminated and FiO_2_ was set by the attending anesthesiologist based on SpO_2_.

### Statistical analysis

All statistical analyses were performed using EZR (version 1.55, Saitama Medical Center, Jichi Medical University, Saitama, Japan), a graphical user interface for R (The R Foundation for Statistical Computing, Vienna, Austria) [8]. For the analysis of data with PaO_2_ < 240 mmHg, Pearson’s correlation coefficient was calculated, and a simple linear regression analysis was performed. A threshold of PaO_2_ < 240 mmHg was chosen, according to previous studies that investigated the relationship between ORi and PaO_2_ [7, 9]. Furthermore, in clinical practice, situations in which PaO_2_ exceeds 240 mmHg are rare. In addition, 95% prediction intervals were calculated for the data of PaO_2_ < 240 mmHg. To determine the optimal cut-off ORi value to detect PaO_2_ ≥ 150 mmHg, receiver operating characteristic (ROC) curve analysis was conducted and the area under the curve (AUC) was calculated to evaluate its diagnostic performance.

To assess the trending ability of ORi, a four-quadrant plot analysis was performed. For this analysis, 60 paired changes in PaO_2_ (ΔPaO_2_) and ORi (ΔORi) were planned to be obtained from 80 datasets (three changes per case). The concordance rate—defined as the percentage of data points in the upper right or lower left quadrant of the four-quadrant plot, where a rate of > 92% is considered good [10]—was calculated. The exclusion zone was defined as the area where the percentage change in ΔORi was < 0.1 and/or PaO_2_ was < 10 mmHg, in accordance with the results of previous studies [7, 11].

## Results

There were two cases in which FiO₂ had already reached 0.21 when it was adjusted to achieve an ORi of approximately 0.5 and could not be reduced further; therefore, four datasets corresponding to the two lower ORi levels could not be obtained. We were unable to identify any specific factors that were unique to these two cases. Therefore, we excluded these two cases and included a total of 76 datasets from 20 patients (12 males and 8 females; mean age, 65 ± 11 years; mean BMI, 24 ± 3 kg/m^2^) in our analysis. The characteristics of these patients are summarized in Table 1.

**Table 1:** Patient characteristics (*n* = 20)

Gender (male/female)	12/8
Age (year)	65 ± 11
Height (cm)	163 ± 10
Weight (kg)	64 ± 13
Body mass index (kg/m^2^)	24 ± 3
ASA physical status	I = 2; II = 18

Age, height, and weight are presented as mean ± standard deviation

*ASA* American society of Anesthesiologists

### ***Relationship between ORi and PaO***_***2***_

**Fig. 2:**
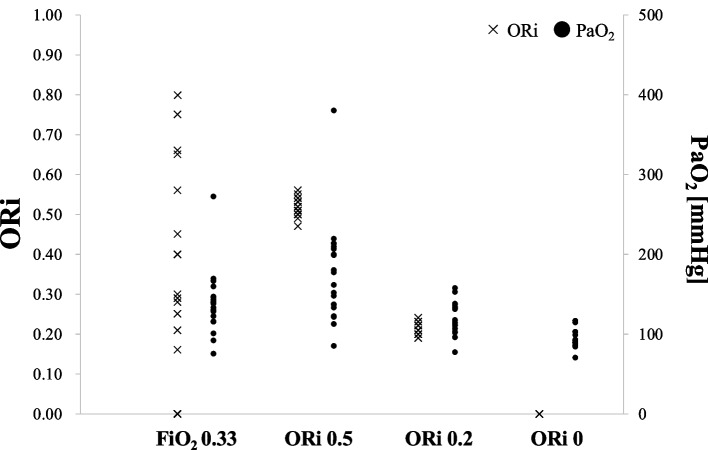
Individual differences in ORi for each case (*n* = 20) at four recording points. “FiO_2_ 0.33” indicates an FiO_2_ setting of approximately 0.33, and “ORi 0.5/0.2/0” indicates ORi values achieved by FiO_2_ adjustment. Black makers show the results of the present study using the updated ORi. *ORi, oxygen reserve index; FiO*_*2*_*, fraction of inspiratory oxygen*

The trends of ORi, PaO_2_, and SpO_2_ in a representative case are shown in Fig. 1. Individual differences in ORi for each case and PaO_2_ at the four recording points are shown in Fig. 2.

**Fig. 3:**
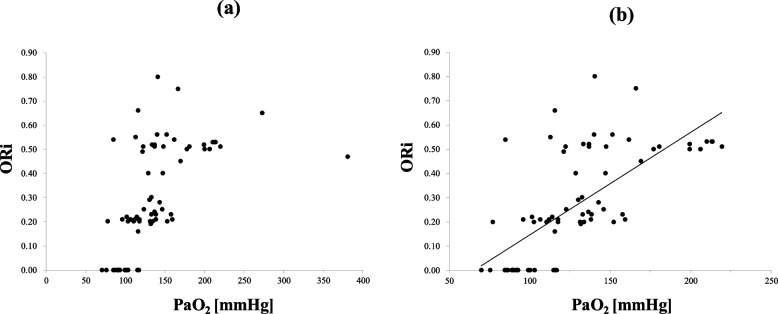
**a** Scatterplot of all ORi (updated version) and PaO_2_ values (*n* = 76). **b** The relationship between ORi and PaO_2_ for PaO_2_ < 240 mmHg (*n* = 73), with 95% prediction intervals. A linear regression analysis (solid line) showed a relatively strong positive correlation (*r*^2^ = 0.4683). *ORi, oxygen reserve index; PaO*_*2*_*, arterial partial pressure of oxygen*

Figure 3a shows the relationship between ORi and PaO_2_ in all 76 datasets, and Fig. 3b shows a scatter diagram of ORi obtained when PaO_2_ < 240 mmHg (73 data sets). Linear regression analysis revealed a relatively strong positive correlation (*r*^2^ = 0.4683) and the resulting regression line is also shown in Fig. 3b.

### Cut-off ORi value for hyperoxemia

**Fig. 4:**
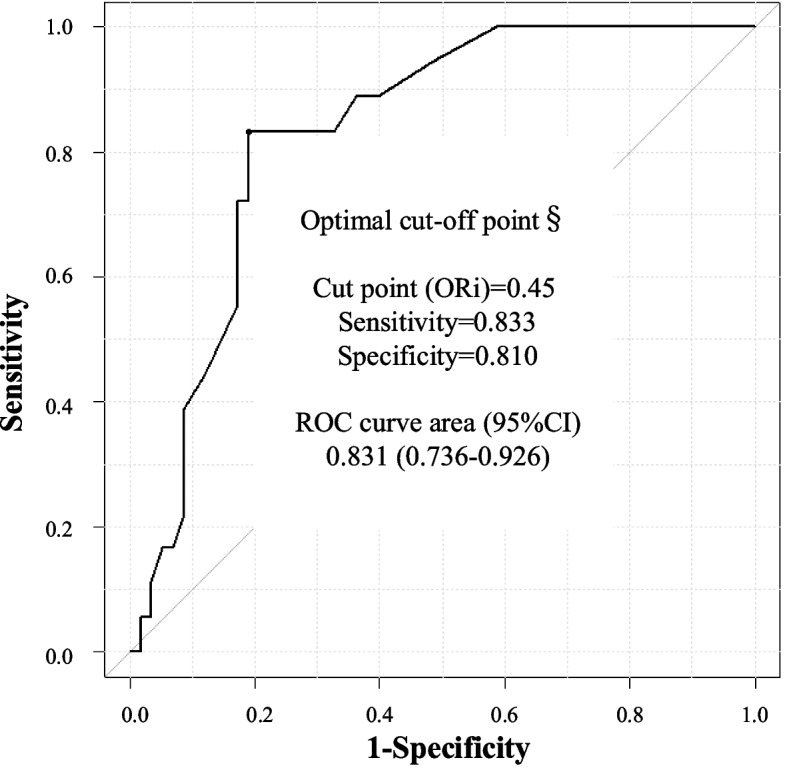
ROC curve analysis to determine the optimal cut-off ORi value for detecting PaO_2_ ≥ 150 mmHg. §: The optimal cut-off point was defined as the point at which the sum of sensitivity and specificity was maximized on the ROC curve. *ROC, receiver operating characteristic; ORi, oxygen reserve index; PaO*_*2*_*, arterial partial pressure of oxygen*

Figure 4 shows the ROC curve used to determine the optimal cut-off ORi value for detecting PaO_2_ ≥ 150 mmHg. The AUC was 0.831 (95% CI, 0.736–0.926), and the cut-off value obtained from the ROC analysis was 0.45 (sensitivity, 0.833; specificity, 0.810).

### ***Correlation between changes in PaO***_***2***_*** and ORi***

**Fig. 5:**
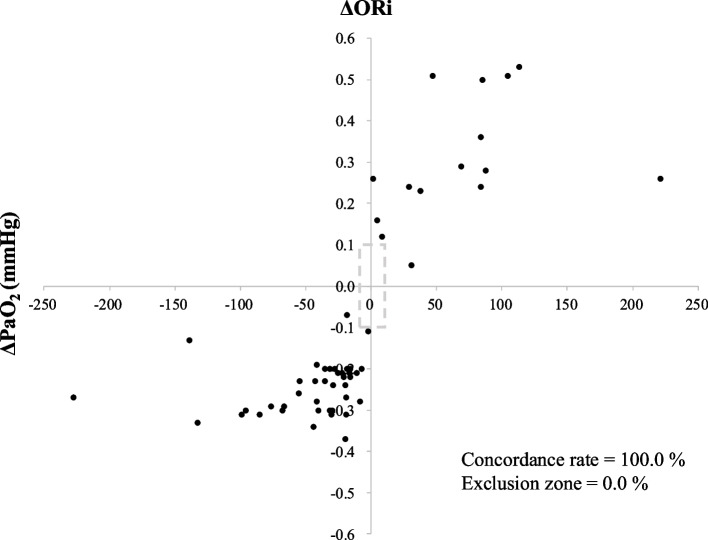
Four-quadrant plot analysis used to assess the trending ability of ΔORi in relation to ΔPaO_2_ (*n* = 56). The area enclosed by the dashed lines represents the exclusion zone, defined as a percentage change in ΔORi < 0.1 and/or PaO_2_ < 10 mmHg. *ORi, oxygen reserve index; PaO*_*2*_*, arterial partial pressure of oxygen*

The four-quadrant plot analysis of 76 datasets to assess the trending ability of ORi revealed a concordance rate of 100.0%, indicating a strong correlation between changes in ORi and PaO₂ (Fig. 5).

## Discussion

The present follow-up study on the relationship between the updated ORi and PaO_2_ revealed that the original and updated versions of ORi demonstrate similar properties regarding their ability to track PaO_2_ changes, but the updated version has a wider absolute value range. Thus, the updated ORi allows for more precise management in the clinical setting, as it more sensitively reflects changes of oxygen reserve in the mild hyperoxemia range compared to the original ORi. In addition, the results of the present study indicate that, although SpO_2_ remained approximately 100% during surgery, ORi fluctuated dramatically in response to changes in FiO_2_ (Fig. 1). Moreover, a four-quadrant plot for assessing the trending ability of ORi also showed a good concordance rate (100.0%). These characteristics of the updated ORi are similar to those of the original ORi measured using Revision L sensor [7].

Compared to the original ORi, which typically ranged from 0.0 to 0.5 [7], the updated version exhibited higher values in the mild hyperoxic range, reaching up to approximately 0.8 under the present study conditions (Fig. 2). The optimal cut-off ORi value to detect PaO_2_ ≥ 150 mmHg was 0.21 in the original ORi (sensitivity, 0.950; specificity, 0.755; AUC, 0.932) [7], but it increased to 0.45 with the updated ORi. As the updated ORi value has more wide range, it may allow more sensitive management of oxygen therapy, especially in the perioperative period for neonates and critically ill patients [12, 13].

Several studies have indicated that differences in ORi versions may affect the results. Early studies demonstrated a linear relationship between ORi and PaO₂ when PaO₂ was < 240 mmHg [7, 9]. However, Ahn et al. investigated FiO_2_ adjustment guided by a combination of SpO_2_ and the updated ORi measured using the Revision O sensor, and reported that their data showed no linear relationship between ORi and PaO_2_ (*r*^2^ = 0.008), even when PaO_2_ was below 240 mmHg (*r*^2^ = 0.015). [10]. Ishida et al. also noted that a plateau ORi value of approximately 1.00 was observed in most cases when using the Radical-7 software version and RD rainbow Lite SET sensor, both released in January 2018. They also reported that the artifact elimination function had been improved compared to the original version [14]. The differences in ORi versions affect the values and their interpretation; thus, the characteristics of each version of ORi should be thoroughly investigated.

With regard to estimating PaO_2_ from ORi, Figs. 2 and 3 demonstrate that even when ORi values are similar, the corresponding PaO₂ values vary widely among patients. Thus, even in the updated ORi, we conclude that we were unable to estimate PaO_2_ from ORi. However, they correlate with PaO_2_, and the ORi trend responded sensitively to changes in FiO₂ and PaO₂ in each individual case. Thus, ORi can be used to noninvasively adjust oxygen concentrations, potentially reducing complications related to hyperoxia or hypoxemia. With future improvements in sensors and software, differences among patients may be reduced, making it possible to adjust oxygen concentrations to specific targets. Kumagai et al. showed that ORi may be useful in titrating postoperative oxygen supplementation without arterial blood sampling [15]. In their study, the supplemental oxygen amount was reduced to 1.0 [0.5–3.0] L/min by targeting an ORi of 0.00. Since their study used the original ORi, further investigations using the updated ORi, including Revision M and O, would be of great interest.

A limitation of the present study is that the patient population differs from that of our previous study using an earlier version of ORi [7], which is no longer available at our hospital following a device update. Therefore, direct comparison between the two versions in the same patients could not be performed and the measurements obtained using each version may have been influenced by differences in patient characteristics rather than by differences between the versions of ORi. However, since the protocol was consistent and patients were selected based on the same inclusion and exclusion criteria, we believe that the results may still be generalizable to some extent.

## Conclusions

We investigated the characteristics of the updated ORi, focusing particularly on the relationship between ORi and PaO_2_. Our findings revealed that the updated ORi displays higher absolute values compared to the original ORi used with Revision L sensor. Further studies are warranted to fully elucidate the characteristics of the new version of ORi and facilitate its effective use in clinical settings. In addition, future publications involving monitoring devices and indices such as ORi should include detailed information on the version.

## Data Availability

Not applicable.

## Abbreviations

ORi: Oxygen reserve index
PaO_2_: Arterial partial pressure of oxygen
SpHb: Percutaneously measured hemoglobin concentration
FiO_2_: Fraction of inspiratory oxygen
SpO_2_: Peripheral oxygen saturation
ROC: Receiver operating characteristic
AUC: Area under the curve
